# Selenium Biofortification Modulates Plant Growth, Microelement and Heavy Metal Concentrations, Selenium Uptake, and Accumulation in Black-Grained Wheat

**DOI:** 10.3389/fpls.2021.748523

**Published:** 2021-10-18

**Authors:** Yuxiu Liu, Shuhua Huang, Zonghao Jiang, Yizhao Wang, Zhengmao Zhang

**Affiliations:** ^1^College of Agronomy, Northwest A&F University, Yangling, China; ^2^College of Horticulture, Northwest A&F University, Yangling, China

**Keywords:** selenium biofortification, selenium ore, black-grained wheat, heavy metals, nutrition

## Abstract

In Se-deficient populations, Selenium- (Se-) enriched wheat is a source of Se supplementation, and Se content can be improved by agronomic biofortification. Thus, black-grained wheat (BGW) and white-grained wheat (WGW) (as the control) were grown in Se naturally contained soils at different concentrations (11.02, 2.21, 2.02, and 0.20 mg·kg^−1^). Then, a field experiment was conducted to assess agronomic performance, the concentration of microelements and heavy metals, and the uptake and distribution of Se in the BGW under the application of Se ore powder. The results showed that the grain yield and grain Se concentration of wheat respectively show a significant increase and decrease from high Se to low Se areas. Higher grain yield and crude protein content were observed in Se-rich areas. The soil application of Se ore powder increased wheat grain yield and its components (biomass, harvest index, grain number, and 1,000 kernels weight). The concentrations of Zn, Fe, Mn, total Se, and organic Se in the grains of wheat were also increased, but Cu concentration was decreased. The concentrations of Pb, As, Hg, and Cr in wheat grains were below the China food regulation limits following the soil application of Se ore powder. Compared with the control, Se ore powder treatment increased the uptake of Se in various parts of wheat plants. More Se accumulation was observed in roots following Se ore powder application, with a smaller amount in grains. In addition, compared with the control, BGW had significantly higher concentrations of Zn, Fe, and Mn and accumulated more Se in grains and shoots and less Se in roots. The results indicate that wheat grown in Se-rich areas increases its grain yield and crude protein content. The soil application of Se ore powder promotes wheat growth and grain yield. Compared with WGW, BGW accumulated more Se in grains and had a higher concentration of organic Se in grains. In conclusion, the application of Se ore powder from Ziyang as Se-enriched fertilizer could be a promising strategy for Se biofortification in the case of wheat, and BGW is the most Se-rich potential genotype.

## Introduction

Selenium (Se) is an essential trace element for humans and is derived primarily from dietary sources (Galic et al., 2021). According to the USDA, the need for human dietary Se intake is 55–200 μg·day^−1^ (Wu et al., 2015). Unfortunately, the estimated Se intake from food consumption in many world regions is below the recommended dose, for example, 19–80 μg·day^−1^ in some populations of New Zealand, 7–11 μg·day^−1^ in the Keshan disease areas (Combs. G. F., 2001) in the developing countries of Asia and Africa (Gupta et al., 2021), and less than 40 μg·day^−1^ in several regions of China (Luo et al., 2021). Se deficiency in the diet is a global health problem (Galic et al., 2021), and it is estimated to be more than 1 billion people worldwide and over 70 million people in China are suffering from Se deficiency (Li et al., 2014; Jones et al., 2017). Se deficiency can adversely affect human health in a number of ways and can cause some severe diseases (Rayman, 2005). Consequently, adequate daily Se intake is essential to maintain human health.

Selenium intake by humans varies according to the diet and Se concentration in the soil in which edible crops are grown (Boldrin et al., 2018). Se levels in soils generally reflect their presence in food crops. Several agricultural areas in the world contain low Se levels in the range of 0.01–2.00 mg·kg^−1^, with a worldwide mean of 0.40 mg·kg^−1^ (Fordyce, 2005). Soils of mainland China have different Se concentrations ranging from 0.01 to 16.24 mg·kg^−1^ with a median of 0.171 mg·kg^−1^ (Liu et al., 2021a). Se concentration in soils of Shaanxi, China was in the range of 0.02–1.67 mg·kg^−1^, with an average of 0.25 mg·kg^−1^ (Liu et al., 2021b). Insufficient Se intake by humans is attributed to general insufficient Se concentration in soils, leading to inadequate Se in food (Jardine and Kidd, 2011). These facts indicate an urgent need to employ Se fortification. Dietary Se fortification can be achieved by dietary diversification, supplements, and food fortification, and biofortification (White, 2016; Boldrin et al., 2018). Among these, the biofortification of edible crops is considered a cost-effective approach to increasing the bioavailable concentration of essential minerals produce by crop genetic or agronomic strategies (White, 2016). Agronomic biofortification refers to the application of fertilizer to the soil, foliar spraying, or the treatment of seeds to improve the status of specific micronutrients in the edible parts of the plant (Sarwar et al., 2020). Previous studies have indicated that agronomic biofortification can increase the amount of available Se (Sarwar et al., 2020). Se biofortification substantially enhances Se concentration in agricultural products and can help alleviate Se malnutrition (Gupta and Gupta, 2007). Cereals are the most common sources of Se in the human diet (Combs. G. F., 2001), and as such represent the most significant plant candidates suitable for Se (Galic et al., 2021).

Wheat (*Triticum aestivum* L.), one of the major cereal crops, is the most important staple food crop to over one-third of the world's population (Reynolds et al., 2012; Boldrin et al., 2018). Wheat has thus become the targeted crop for Se biofortification, with additional possible positive effects on grain yield. Se-enriched wheat has long been considered as a source of Se supplementation for Se-deficient populations (Finley, 2005). Unfortunately, grain Se concentration in the wheat produced in China ranges from 0 to 821.0 μg·kg^−1^ with an average of 64.6 μg·kg^−1^, and 63% of the wheat produced is Se- deficient although much of the population depends primarily on wheat-derived foods for Se (Liu et al., 2016b). Se concentration in the grains of wheat can be attributed to crop husbandry, Se fertilization, and weather conditions (Gupta et al., 2021). Wheat has a relatively high ability to transport Se from the straw to grain (Wang et al., 2017) although there is a relatively small genetic variation concerning the ability to accumulate Se in the whole plant (Hawkesford and Zhao, 2007). Thus, Se biofortification in wheat *via* agronomic approaches has been shown to be practical. It has been demonstrated that agronomic biofortification provides an effective approach to increasing Se concentration in wheat (Hawkesford and Zhao, 2007; Boldrin et al., 2018; Xia et al., 2020; Galic et al., 2021; Gupta et al., 2021; Wang et al., 2021). Se-enriched wheat grains can be used as a dietary source of Se to reduce the occurrence of Se deficiency-related health problems. However, excess accumulation is toxic to both humans and plants. For higher plants, Se at low concentrations enhances crop growth and stress tolerance, but Se at high concentrations leads to a drastic reduction in yield (Gupta and Gupta, 2007). Therefore, to avoid excess accumulation of Se, it is necessary to identify the most effective and appropriate Se concentration in fertilizers.

Moreover, the beneficial or toxic effects of Se not only depend on concentration in fertilizers but also are related to the form of Se available in the soil solution (Boldrin et al., 2018). Selenite (Se^4+^) and selenate (Se^6+^) are the major Se forms for plant absorption and utilization, and the absorption levels of Se^4+^ and Se^6+^ are 50 and 100%, respectively (Gupta et al., 2021). A sodium selenate (Na_2_SeO_4_) applied through roots increased Se content in the plant tissues of wheat in both shoots and grains (Boldrin et al., 2018). Soil and foliar applications *via* sodium selenite (Na_2_SeO_3_) enhanced organic grain Se concentration within wheat (Xia et al., 2020). Both Na_2_SeO_4_ and Na_2_SeO_3_ soil application increased action on transporters, thereby affecting the accumulation of Se in wheat (Wang et al., 2021). Ziyang, located in the Daba Mountain area of Southern Shannxi Province, China, is a well-known Se-enriched area, with Se distribution in rocks ranging from 0.23 to 57.0 mg·kg^−1^ (Tian et al., 2016b). Crops grown in areas around the Se-rich core are enriched in Se, and Ziyang has become the leading area of the Se industry. Se in Se ore powder is mainly present as Se^4+^, and elemental Se (Se°) is formed at a 4:6 ratio (Deng et al., 2018). Se^4+^ is the most common species of Se fertilizers, suggesting that Se ore powder can be potentially absorbed and utilized by plants. Moreover, Pb, As, Hg, and Cr concentrations in brown rice and soybean seeds were below the limits of detection, and the organic Se accounted for over 96% of the total Se in both crops following the soil application of Se ore powder (Deng et al., 2018). Therefore, reasonable selection and the use of Se-enriched fertilizers could provide an effective approach for the biofortification of wheat for Se, and thereby solving the health problem caused due to Se deficiency. Determining the potential to accumulate Se in the different parts of wheat plants is important for the success of biofortification programs.

An increase in the wheat yield potential in the past few decades has been accompanied by a decrease in grain Se (Garvin et al., 2006). This result has necessitated the breeding of wheat cultivars with a high Se level. The accumulation of Se in grains varies significantly among different cultivars or pre-breeding and advanced lines (Gupta et al., 2021). However, limited germplasm has been screened for Se, compared with Zn and Fe. Color-grained wheat provides an opportunity to select Se-enriched genotypes. Color-grained wheat is characterized by its grains of different colors, including black, blue, and green, in comparison with common wheat (white or red) (Liu et al., 2021c). Color-grained wheat has significant levels of anthocyanins and essential nutrients (Zn and Fe) that are beneficial to human health and has the potential to produce value-added and functional flour products (Saini et al., 2020). Protein and micronutrient contents in some of the Chinese black-grained wheat (BGW) lines were higher than those in some of the white-grained wheat (WGW) cultivars (Li et al., 2006; Liu et al., 2021c). The production of safe Se-enriched agricultural products can be achieved by Se ore powder application (Deng et al., 2018). It is thus necessary to determine whether the Se concentration of BGW is higher both when grown in soils with naturally abundant Se and grown under controlled Se conditions.

The objectives of this study were to (1) investigate the grain yield, grain Se concentration, and nutritional components of BGW grown in naturally high Se, Se-rich, and low Se areas; (2) analyze the effect of Se ore powder application through the soil on grain yield and its components and microelements and to assess the safety of the edible parts of BGW and investigate its effect on the uptake and distribution of Se in wheat; and (3) make comparisons of such an effect between BGW and WGW (as the control) to assess agronomic performances and the uptake ability of BGW.

## Materials and Methods

### Plant Materials

Four wheat genotypes were selected for this study, consisting of two BGW genotypes [Xihei 88 (BGW-1) and Heidali (BGW-2)] and two WGW cultivars [Chuanmai 37 (WGW-1) and Pubing 151 (WGW-2)]. WGW cultivars were set as the controls. WGW-1 is suitable for planting in southern Shaanxi and a check variety, whereas WGW-2 is suitable in dry land on the Weibei dryland plateau and similar ecological regions in Shaanxi. These plant materials have been bred and/or preserved in the Prof. Zhang' Lab, College of Agronomy, Northwest A&F University, Shaanxi, China.

### Experimental Design

The study consisted of two parts.

In the first part of this study, we investigated grain yield, crude protein content, ash content, crude fat, and Se concentration in the grains of wheat when planted in different Se-containing soils in Ziyang, Shaanxi, China. The annual on-site precipitation was ~1,100 mm. The first experiment was performed in the 2015–2016 growing season (October to June). BGW-1, BGW-2, and WGW-1 were planted in the four Se areas: a high Se area [Se concentration in the soil of 11.02 mg·kg^−1^, Shuanghekou Village, Shuang‘an, Ziyang, China (32°41′88″N, 108°28′32″W, altitude 386 m)], Se-rich area 1 [2.21 mg·kg^−1^, Qingfeng Village, Hengkou, Ankang, China (32°43′85″N, 108°47′75″W, altitude 278 m)], Se-rich area 2 [2.02 mg·kg^−1^, Wulangping Village, Hanwang, Ziyang, China (32°75′76″N, 108°48′72″W, altitude 432 m)], and a low-Se area [0.20 mg·kg^−1^, Yangling, Shaanxi, China (34°15′3″N, 108°1′48″W, altitude 408 m)]. Organic matter content, carbonate content, and pH for the 0–20 cm soil layers were in the range of 21.7–25.3, 194.5–207.7, and 7.21–7.45 g·kg^−1^, with an average of 23.40 ± 1.81, 201.63 ± 6.68, and 7.50 ± 0.32 g·kg^−1^ in high Se area, Se-rich area 1, and Se-rich area 2, respectively, according to Bao (2000). There were no significant differences in organic matter content, carbonate content, and pH among high Se and Se-rich areas. The experimental plots were arranged in a randomized complete block design with three replicates. Plots were 10.0 × 1.32 m^2^, with six rows and a spacing of 0.22 m between rows. Wheat seeds were sown at a density of 150 kg·ha^−1^, and 750 kg·ha^−1^ of ammonium bicarbonate (N, 16%), and 150 kg·ha^−1^ of diamine Phosphate (N, 18%; P_2_O_5_, 46%) were applied for the 0–20 cm soil layers prior to planting. Weed control was performed by hand. No additional fertilizers or pesticides were applied during plant growth. The sowing date was October 28, 2015, and the plants were harvested on June 5, 2016.

In the second part of this study, a completely randomized two-factor (Se application rate and wheat genotype) design with three replicates was used to examine the effect of Se ore powder on grain yield and its components, the absorption of Zn, Fe, Mn, Cu, Pb, As, Hg, and Cr, and the uptake and accumulation of Se in wheat. The field experiment was conducted at the research farm of Northwest A&F University, Yangling, Shaanxi, China (34°17′38″N, 108°4′23″W, elevation 525 m) in the 2018–2019 and 2019–2020 growing season. Each plot was 9.0 × 1.2 m^2^, with six rows and 0.20 m between the rows. According to the Soil Physicochemical Analysis Handbook (Bao, 2000), the soil is classified as Loess loam, with an organic matter content of 9.7 g·kg^−1^, total N content of 1.5 g·kg^−1^, the available P content of 10.5 mg·kg^−1^, the available K content of 250.1 mg·kg^−1^, total Se of 0.24 mg·kg^−1^, and pH of 8.2 for the 0–20 cm soil layers at the seeding of a wheat field.

BGW-1, BGW-2, and WGW-2 were used in the second part of this study. Wheat seeds were planted at a density of 280 seeds per m^2^ using a planting machine [2BZL-6 (A)]. Se ore powder with Se concentration of 51.3 mg·kg^−1^ was used as the Se fertilizer. Se ore rocks were collected from Naore Village, Shuang'an, Ziyang, Shaanxi, China, then cleaned with tap water to wipe off all contaminants, washed with deionized water, and air-dried and powdered to 0.074 mm using a Retsch RS300XL grinding mill (Tian et al., 2016b). Five Se application levels (pure Se) were set as with no Se fertilizer (CK), 1,080 (T1), 2,160 (T2), 3,240 (T3), and 4,320 g·ha^−1^ (T4) and were applied to the field before plowing according to the method of Liu et al. (2016a). Se concentrations in soil were determined to be 0.24 ± 0.04, 0.65 ± 0.05, 1.04 ± 0.04, 1.45 ± 0.06, and 1.86 ± 0.05 mg·kg^−1^, respectively, before planting. Before sowing, 600 kg·ha^−1^ of compound fertilizer (20-20-6, Summit Fertilizer (Qingdao) Co., Ltd., Shandong, China) containing N 20%, P_2_O_5_ 20%, and K_2_O 5% was applied to the 0–20 cm layers.

**Figure 1:**
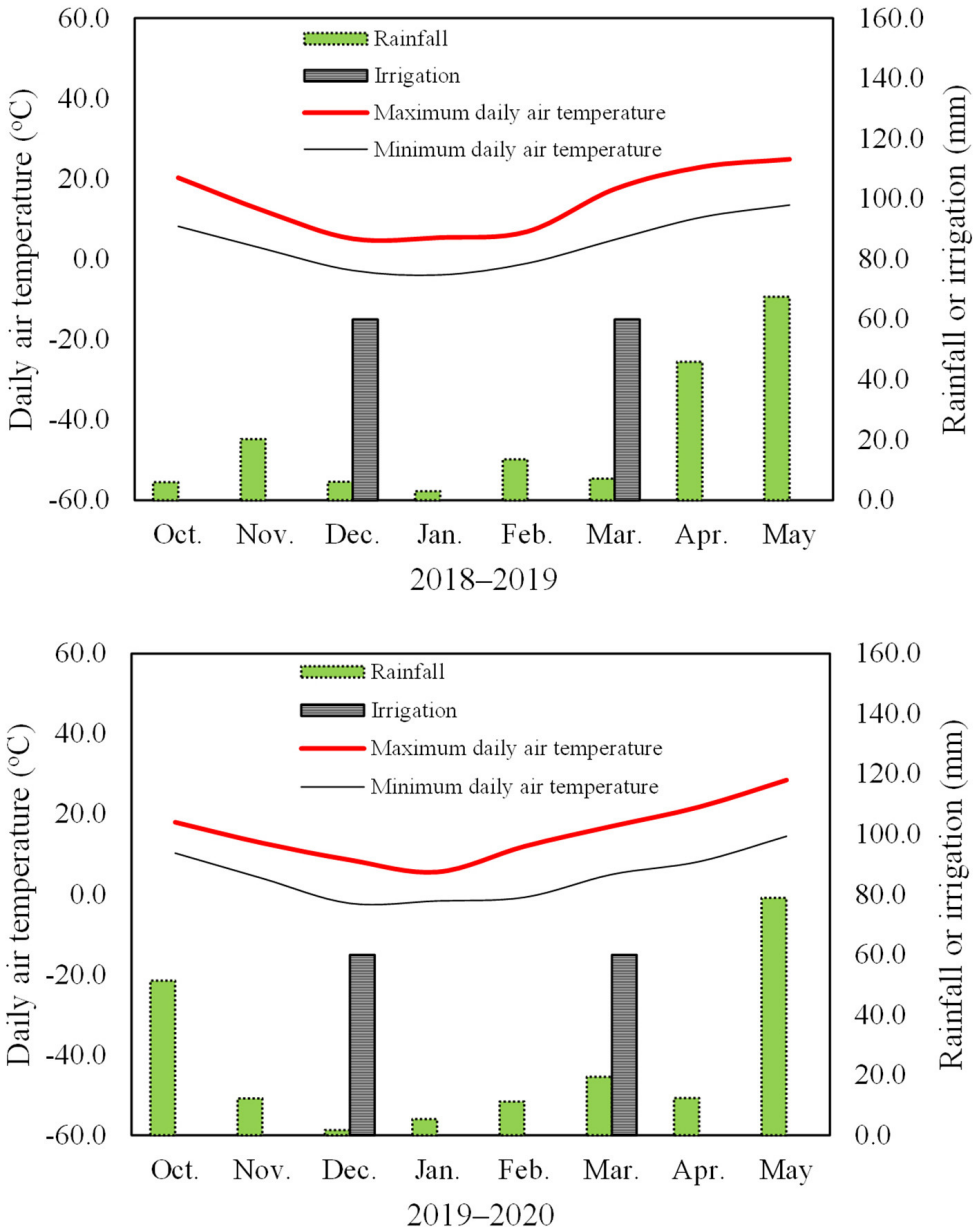
Maximum and minimum daily air temperatures at 2 m, rainfall amount, and irrigation in the two wheat growing seasons.

The rainfall from October to May was 169.4 and 192.5 mm in the two growing seasons (Figure 1). Irrigation at 1,200 m^3^/ha (120 mm irrigation) was provided using tap water at two times, in the wintering stage (600 m^3^/ha, December 30, 2018, and 2019) and in the greening stage (600 m^3^/ha, March 15, 2018, and 2019) in each growing season. The maximum and minimum daily air temperatures at the experimental site are shown in Figure 1. Herbicides, fungicides, and insecticides were applied as required during the experiment. The seeds were sown on October 5, 2018, and October 7, 2019, and the plants were harvested on June 2, 2019, and June 5, 2020.

### Measurement of Grain Yield and Its Nutritional Components

At the maturity stage (Feekes 11.3–11.4) according to Miller (1999), plots were harvested by hand in the first part of this study and by small-plot combines (4LXNK-1.0, Weihai Xinnongke Machinery Factory, Shandong, China) in the second part of this study. Grain yield (t·ha^−1^) was determined from the harvested weight in a plot adjusted to 12% moisture content. Plants were sampled by cutting them from the soil surface in a 1 m^2^ plot with four rows for biomass and yield components. The samples of each plot were air-dried at 50°C in the greenhouse to a constant weight and weighted to obtain biomass (t·ha^−1^). Harvest index (%) was calculated as the ratio of grain yield relative to biomass. Spike numbers (m^2^) were measured from the plant samples and then transformed to spike number per m^2^. Grain number (spike^−1^) was determined as the average of 10 spikes randomly selected from the harvested samples. About 1,000 kernel weight (g) was measured using 1,000 grains from the harvested samples in each plot.

Crude protein content (g·100 g^−1^) was measured according to the Kjeldahl nitrogen determination method (the Standard Method GB 5009.5-2010 developed by the Ministry of Health of China) with an automatic nitrogen determination analyzer (Kjeltec 8400, FOSS). Crude fat content (g·100 g^−1^) was evaluated by the Standard Method GB/T 5009.6-2003 developed by the Ministry of Health of China. Ash content (g·100 g^−1^) was determined by the dry combustion of 3 g of the sample in a muffle furnace (Jujing, Shanghai, China) at 580°C for 16 h according to the Standard Method GB 5009.4-2010 developed by the Ministry of Health of China.

### Measurement of Zn, Fe, Mn, Cu, Se, Pb, As, Hg, and Cr Concentrations

Whole wheat plants were harvested, washed thoroughly with tap water and distilled water three times, and then dried with tissue paper. Grain, spike-stalk + glum, leaves, stem + leaf sheath, and root were separated and dried in an air-forced oven at 75°C to a constant weight. The oven-dried samples were ground using a freezing mixer mill (MM400, Retsch, Haan, Germany) through 100-mesh sieves, and then stored in a sealed plastic bag for the measurement of Zn, Fe, Mn, Cu, Se, Pb, As, Hg, and Cr concentrations (mg·kg^−1^). The microelement and heavy metal element concentrations of samples were determined by quantifying the aqueous constituents following microwave digestion with HNO_3_-H_2_O_2_ solution. Blank and standards [GBW(E) 080215 for Se, GNM-M22809-2013 for other elements] were included for quality assurance.

A 0.5-g pulverized sample was transferred to a clear polytetrafluoroethylene digestion tube and then was added with 10.0 ml HNO_3_ and 2.0 ml H_2_O_2_. The sample was digested in a closed microwave digestion system (MARS6, CEM, Matthews, NC, USA). After cooling, the digestion solution was transferred to a 25-ml volumetric flask, diluted with ultrapure water to a set volume, and mixed for the measurement of Zn, Fe, Mn, Cu, Pb, As, Hg, and Cr concentrations according to the Standard Method GB/T 5009.14-2017 developed by the Ministry of Health of China using atomic absorption spectrometry (AAS) (PinAAcle 900F, PerkinElmer Enterprise Management Co., Ltd., Waltham, MA, USA).

About 5 ml of HCl (6.0 mol·L^−1^) was added to the digestion solution after cooling to room temperature. The digestion solution was then diluted with ultrapure water to 25.0 ml, and 10 ml of the solution was transferred to a reaction vessel, with 2.5 ml K_3_Fe (CN)_3_ (100 g·L^−1^) being added for subsequent total Se concentration measurement according to the Standard Method GB/T 5009.93-2017 developed by the Ministry of Health of China using a liquid-phase atomic fluorescence spectrometer (LC-AFS9780, Beijing Haiguang Instrument Co., Ltd., Beijing, China).

Firstly, inorganic Se concentration in grains was measured. A 1.0-g pulverized sample was weighed and transferred into a test tube with a cork, with 5.0 ml HCl (w/w) (50%) being subsequently added. The solution was mixed for 30 min and placed in a 100°C water bath for 30 min. After cooling, the solution was then filtered with absorbent filter paper, and the filtrate was subjected to the total Se content determination protocol.


$$\begin{array}{l} \text{Organic Se content}(mg\cdot \;kg^{-\text{1}})=\text{Total Se content}\\ \;-\text{Inorganic Se content}. \end{array}$$

### Measurement of Se Distribution and Accumulation

Selenium distribution and accumulation in each part were measured as follows:


$$\begin{array}{l} {\text{D}}_{Se}\;=\;\frac{{\text{C}}_{i}\times D_{w}}{A_{w}} \end{array}$$

where D_*Se*_ is the proportion of Se distributed in each part of the wheat plant (%), *C*_*i*_ is the Se concentration in the grain, spike-stalk + glum, leaves, stem + sheath, and root (mg·kg^−1^); *D*_*w*_ is the dry weight of the grain, spike-stalk + glum, leaves, stem + sheath, and root (kg·plant^−1^); and *A*_*w*_ is Se concentration in the wheat plant (mg·plant^−1^).

The translocation factor of Se from the root to grain (TF_root−grain_) was measured as follows:


$$\begin{array}{l} {\text{TF}}_{\text{root}-\text{grain}}=\;\frac{C_{\text{grain}}}{C_{\text{root}}} \end{array}$$

where *C*_grain_ is Se concentration in the grain (mg·kg^−1^) and *C*_root_ is Se concentration in the root (mg·kg^−1^).

### Statistical Analysis

Significant differences for the measured traits were detected by ANOVA procedures in the JMP V12.0 statistical software from SAS (version 9, SAS Institute, Inc., Cary, NC, USA). The data were expressed as mean values ± SD. Significant differences between different Se application levels as well as between BGW and WGW genotypes were detected using Fisher's protected LSD at α = 0.01 and α = 0.05, respectively.

## Results

### Grain Yield, Grain Se Concentration, and Nutritional Components of Wheat in Different Se Areas

As indicated in Table 1, the grain yield and grain Se concentration of wheat respectively show a significant increase and decrease from high Se to low Se areas, and its highest value was observed in the Se-rich area 2 (4.49–5.28 t·ha^−1^) and high Se area (4.05–5.23 mg·kg^−1^) (α = 0.01). Wheat had significantly higher crude protein content when grown in Se-rich areas compared to other areas but had the lowest crude protein content in the high Se area (α = 0.01). However, there were no significant differences in the crude fat content or ash content of wheat between under high Se and Se-rich areas.

**Table 1:** Performance of the grain yield, grain selenium (Se) concentration, and nutritional components of wheat in different Se areas.

**Items**	**Areas**	**BGW-1**	**BGW-2**	**WGW-1**
Grain yield (t·ha^−1^)	High Se area	3.20 ± 0.19^Ca^	3.03 ± 0.22^Ca^	3.39 ± 0.16^Ca^
	Se-rich area 1	4.23 ± 0.18^Ba^	3.66 ± 0.19^Bb^	4.35 ± 0.16^Ba^
	Se-rich area 2	5.16 ± 0.17^Aa^	4.49 ± 0.18^Ab^	5.28 ± 0.13^Aa^
	Low Se area	-	-	-
				
Se (mg·kg^−1^)	High-Se area	5.23 ± 0.08^Aa^	4.43 ± 0.08^Ab^	4.05 ± 0.04^Ac^
	Se-rich area 1	1.44 ± 0.07^Ba^	1.08 ± 0.06^Bb^	1.01 ± 0.05^Bb^
	Se-rich area 2	1.31 ± 0.06^Ba^	1.15 ± 0.07^Ba^	0.85 ± 0.03^Bb^
	Low-Se area	0.04 ± 0.00^Ca^	0.05 ± 0.00^Ca^	0.04 ± 0.00^Ca^
				
Crude protein content	High-Se area	15.34 ± 0.07^Da^	12.76 ± 0.07^Db^	11.46 ± 0.06^Dc^
(g·100^−1^)	Se-rich area 1	20.24 ± 0.05^Aa^	18.65 ± 0.06^Ab^	17.25 ± 0.06^Ac^
	Se-rich area 2	18.93 ± 0.06^Ba^	16.35 ± 0.04^Bb^	15.46 ± 0.08^Bc^
	Low-Se area	17.25 ± 0.08^Ca^	14.32 ± 0.05^Cb^	13.06 ± 0.07^Cc^
				
Crude fat content	High-Se area	2.21 ± 0.05^Aa^	2.18 ± 0.20^Aa^	2.08 ± 0.10^Aa^
(g·100^−1^)	Se-rich area 1	2.08 ± 0.04^Aa^	2.09 ± 0.07^Aa^	1.92 ± 0.08^Aa^
	Se-rich area 2	2.03 ± 0.07^Aa^	2.05 ± 0.10^Aa^	1.85 ± 0.07^Aa^
	Low-Se area	1.87 ± 0.22^Aa^	1.82 ± 0.08^Aa^	1.78 ± 0.06^Aa^
				
Ash content (g·100^−1^)	High-Se area	1.63 ± 0.01^Aa^	1.58 ± 0.01^Aa^	1.56 ± 0.01^Aa^
	Se-rich area 1	1.60 ± 0.01^Aab^	1.68 ± 0.01^Aa^	1.53 ± 0.01^Ab^
	Se-rich area 2	1.67 ± 0.02^Aa^	1.62 ± 0.01^Aa^	1.60 ± 0.02^Aa^
	Low-Se area	1.41 ± 0.05^Aa^	1.26 ± 0.02^Bab^	1.21 ± 0.04^Bb^

*Different capital letters in the same column indicate a significant difference at α = 0.01. Different lowercase letters in the same row indicate a significant difference at α = 0.05. BGW-1, black-grained wheat genotype Xihei 88; BGW-2, black-grained wheat genotype Heidali; WGW-1, white-grained wheat cultivar Chuanmai 37*.

Grain yield, grain Se concentration, and nutritional components were compared between BGW (BGW-1 and BGW-2) and WGW (WGW-1) grown in different Se areas (Table 1). No significant differences were observed for grain yield among wheat genotypes in the high Se area. BGW-2 had a significantly lower grain yield than WGW-1 in Se-rich areas (α = 0.05), but the grain yield of BGW-1 did not significantly differ from that of WGW-1. BGW-1 had a significantly higher Se concentration in grains than WGW-1 in high Se and Se-rich areas (α = 0.05), but no significant differences were found between the two genotypes in the low Se area. The highest crude protein content was significantly observed for BGW-1 in the four Se areas (α = 0.05), but no significant differences were observed for crude fat content among wheat genotypes. There were no significant differences in ash content between BGW-1 and WGW-1 in high Se and Se-rich areas.

### Effect of Se Application on the Grain Yield and Yield Component of Wheat

The highest grain yield, biomass, and harvest index were observed for wheat under T2 treatments (2,160 g·ha^−1^ pure Se) among all treatments in the two seasons (α = 0.01), but no significant differences were observed under T1 (1,080 g·ha^−1^ pure Se) and T3 (3,240 g·ha^−1^ pure Se) treatments (Table 2). The soil application of Se ore powder had no significant effect on spike numbers of wheat in either season. Grain number and 1,000 kernels weight of wheat were the highest under T2 among the treatments.

**Table 2:** Effects of Se fertilization on the grain yield and yield components of wheat in the two seasons.

**Seasons**	**Treatments**	**Grain yield (t·ha^−1^)**			**Biomass (t·ha^−1^)**			**Harvest index (%)**		
		**BGW-1**	**BGW-2**	**WGW-2**	**BGW-1**	**BGW-2**	**WGW-2**	**BGW-1**	**BGW-2**	**WGW-2**
2018–2019	T0	5.29 ± 0.22^Ca^	4.56 ± 0.13^Cb^	5.33 ± 0.13^Ca^	17.2 ± 0.40^Ca^	16.2 ± 0.38^Bb^	17.3 ± 0.31^Ba^	30.7 ± 0.62^Ca^	28.2 ± 0.30^Cb^	30.8 ± 0.25^Ca^
	T1	5.84 ± 0.12^Ba^	5.07 ± 0.18^Bb^	5.89 ± 0.13^Ba^	18.6 ± 0.35^ABa^	17.1 ± 0.52^ABb^	18.2 ± 0.30^ABa^	31.5 ± 0.32^BCc^	29.6 ± 0.35^Bb^	32.3 ± 0.31^Ba^
	T2	6.52 ± 0.13^Aa^	5.56 ± 0.10^Ab^	6.43 ± 0.14^Aa^	19.5 ± 0.57^Aa^	18.1 ± 0.46^Ab^	19.2 ± 0.49^Aa^	33.5 ± 0.35^Aa^	30.7 ± 0.20^Ab^	33.4 ± 0.20^Aa^
	T3	5.90 ± 0.21^Ba^	5.10 ± 0.23^Bb^	5.82 ± 0.18^Ba^	18.5 ± 0.40^ABa^	17.3 ± 0.50^ABb^	18.0 ± 0.38^Bab^	31.9 ± 0.40^Ba^	29.5 ± 0.61^Bb^	32.4 ± 0.36^Ba^
	T4	5.75 ± 0.17^Ba^	4.90 ± 0.14^BCb^	5.74 ± 0.22^BCa^	18.1 ± 0.56^BCa^	16.8 ± 0.40^Bb^	18.1 ± 0.55^Ba^	31.7 ± 0.25^BCa^	29.1 ± 0.21^BCb^	31.8 ± 0.21^Ba^
2019–2020	T0	5.93 ± 0.27^Ca^	4.79 ± 0.19^Cb^	5.90 ± 0.12^Ca^	19.0 ± 0.42^Ca^	16.8 ± 0.55^Cb^	19.0 ± 0.53^Ba^	31.3 ± 0.76^Ca^	28.5 ± 0.35^Cb^	31.0 ± 0.32^Ca^
	T1	6.68 ± 0.17^Ba^	5.31 ± 0.15^Bb^	6.51 ± 0.17^Ba^	20.3 ± 0.21^Ba^	17.7 ± 0.36^BCb^	20.0 ± 0.21^ABa^	32.9 ± 0.55^ABa^	30.0 ± 0.26^Bb^	32.5 ± 0.50^Ba^
	T2	7.28 ± 0.20^Aa^	5.99 ± 0.19^Ab^	7.24 ± 0.25^Aa^	21.4 ± 0.35^Aa^	18.9 ± 0.25^Ab^	21.2 ± 0.55^Aa^	34.1 ± 0.45^Aa^	31.8 ± 0.59^Ab^	34.2 ± 0.41^Aa^
	T3	6.52 ± 0.12^Ba^	5.34 ± 0.20^Bb^	6.48 ± 0.22^Ba^	20.1 ± 0.26^Ba^	17.9 ± 0.46^ABb^	19.8 ± 0.47^Ba^	32.5 ± 0.21^BCa^	29.8 ± 0.50^Bb^	32.6 ± 0.40^Ba^
	T4	6.35 ± 0.28^BCa^	5.22 ± 0.10^BCb^	6.29 ± 0.14^BCa^	19.8 ± 0.49^BCa^	17.6 ± 0.26^BCb^	19.7 ± 0.40^Ba^	32.0 ± 0.61^BCa^	29.6 ± 0.15^Bb^	32.0 ± 0.35^BCa^
		**Spike number (m** ^**2**^ **)**			**Grain number (spike^−1^)**			**Thousand kernels weight (g)**		
2018–2019	T0	312.0 ± 13.9^Ac^	385.1 ± 13.0^Ab^	437.3 ± 16.7^Aa^	63.9 ± 1.25^Ba^	43.4 ± 0.76^Bb^	42.7 ± 0.67^Bb^	39.2 ± 0.51^Bb^	38.9 ± 0.98^Bb^	41.3 ± 0.89^Ba^
	T1	310.7 ± 15.1^Ac^	400.0 ± 16.0^Ab^	449.7 ± 18.3^Aa^	66.2 ± 1.21^ABa^	46.7 ± 1.55^ABb^	44.2 ± 0.69^ABc^	41.8 ± 0.57^Ab^	39.9 ± 1.01^ABc^	43.6 ± 0.71^ABa^
	T2	332.0 ± 14.4^Ac^	410.3 ± 10.2^Ab^	468.6 ± 9.97^Aa^	67.7 ± 0.93^Aa^	47.4 ± 1.50^Ab^	45.8 ± 0.79^Ab^	42.7 ± 1.30^Aab^	42.0 ± 0.85^Ab^	44.0 ± 0.87^Aa^
	T3	313.2 ± 14.2^Ac^	394.7 ± 12.2^Ab^	446.2 ± 10.9^Aa^	66.5 ± 1.10^ABa^	46.3 ± 1.49^ABb^	44.2 ± 0.85^ABb^	41.6 ± 0.65^Aab^	41.1 ± 1.20^ABb^	43.4 ± 1.17^ABa^
	T4	312.1 ± 15.8^Ac^	392.0 ± 13.9^Ab^	443.5 ± 12.0^Aa^	65.3 ± 0.87^ABa^	45.7 ± 1.15^ABb^	43.9 ± 0.55^Bb^	41.5 ± 1.15^ABab^	40.3 ± 1.21^ABb^	43.3 ± 0.95^ABa^
2019–2020	T0	344.0 ± 8.00^Ac^	416.0 ± 8.00^Ab^	472.0 ± 13.9^Aa^	64.0 ± 1.59^Ba^	44.3 ± 1.81^Bb^	44.1 ± 1.03^Bb^	39.6 ± 0.95^Bb^	38.2 ± 0.68^Cb^	41.7 ± 0.64^Ba^
	T1	352.0 ± 8.00^Ac^	429.3 ± 4.62^Ab^	485.3 ± 12.2^Aa^	66.1 ± 1.42^ABa^	46.3 ± 1.33^ABb^	46.0 ± 1.06^ABb^	42.2 ± 0.86^ABa^	39.3 ± 0.61^BCb^	42.9 ± 0.83^ABa^
	T2	365.3 ± 4.62^Ac^	437.3 ± 16.0^Ab^	496.0 ± 16.0^Aa^	68.2 ± 1.00^Aa^	48.3 ± 1.22^Ab^	48.3 ± 1.45^Ab^	42.3 ± 0.94^Aab^	41.5 ± 0.85^Ab^	44.4 ± 0.81^Aa^
	T3	349.3 ± 12.2^Ac^	421.3 ± 4.60^Ab^	488.0 ± 13.8^Aa^	66.0 ± 1.20^ABa^	46.0 ± 1.40^ABb^	45.1 ± 1.42^ABb^	41.6 ± 1.27^ABab^	40.5 ± 0.50^ABb^	43.3 ± 0.64^ABa^
	T4	346.7 ± 9.24^Ac^	418.7 ± 16.7^Ab^	480.0 ± 16.0^Aa^	65.1 ± 1.22^ABa^	45.4 ± 1.56^ABb^	44.3 ± 1.21^Bb^	41.4 ± 1.04^ABb^	40.4 ± 0.66^ABb^	43.5 ± 1.29^ABa^

*Different capital letters in the same column indicate a significant difference at α = 0.01. Different lowercase letters in the same row indicate a significant difference at α = 0.05. T0, no Se fertilizer; T1, 1,080 g·ha^−1^ pure Se; T2, 2,160 g·ha^−1^ pure Se; T3, 3,240 g·ha^−1^ pure Se; T4, 4,320 g·ha^−1^ pure Se; BGW-1, black-grained wheat genotype Xihei 88; BGW-2, black-grained wheat genotype Heidali; WGW-2, white-grained wheat cultivar Pubing 151*.

The effects of Se application on grain yield and its components were investigated between BGW (BGW-1 and BGW-2) and WGW (WGW-2) genotypes under different treatments (Table 2). The grain yield, biomass, and harvest index of BGW-2 were significantly lower than those of WGW-2 under all treatments in both seasons (α = 0.05), but no significant differences were observed between BGW-1 and WGW-2. BGW had a significantly lower spike number than WGW-2 under all treatments in both seasons, and the lowest spike number was observed for BGW-1 (α = 0.05). The grain number of BGW-1 seemed to be the significantly highest among all wheat genotypes under five treatments in both seasons (α = 0.05), but no significant differences were found between BGW-2 and WGW-2. WGW-2 had the highest 1,000 kernels weight among wheat genotypes under the five treatments in both seasons.

### Effect of Se Application on Microelement Concentrations in the Grains of Wheat

As shown in Table 3, the soil application of Se ore powder increased Zn, Fe, and Mn concentrations in the grains of wheat relative to T0 (no Se fertilizer) in both seasons, but decreased Cu concentration. The highest Zn and Mn concentration was found in the grains of wheat under T3 treatments, whereas the highest Fe concentration was observed under T2 treatments (α = 0.01). There were no significant differences for Cu concentration in the grains of wheat among T0, T1, T2, and T3 treatments in either of the seasons.

**Table 3:** Effects of Se fertilization on Zn, Fe, Mn, and Cu concentrations in the grains of wheat in the two seasons.

**Seasons**	**Treatments**	**Zn (mg·kg^−1^)**			**Fe (mg·kg^−1^)**		
		**BGW-1**	**BGW-2**	**WGW-2**	**BGW-1**	**BGW-2**	**WGW-2**
2018–2019	T0	35.90 ± 1.17^Ba^	33.19 ± 1.28^Ca^	29.36 ± 1.31^Cb^	33.11 ± 0.96^Cb^	37.09 ± 1.00^Ca^	29.47 ± 0.94^Cc^
	T1	38.64 ± 0.86^Ba^	35.08 ± 1.33^BCb^	32.02 ± 0.99^BCc^	40.26 ± 1.13^Ba^	39.47 ± 0.81^BCa^	33.58 ± 1.16^Bb^
	T2	43.39 ± 1.39^Aa^	38.11 ± 1.06^ABb^	34.65 ± 1.02^ABc^	47.21 ± 1.15^Aa^	44.54 ± 1.07^Aa^	37.75 ± 1.20^Ab^
	T3	46.04 ± 1.03^Aa^	42.11 ± 1.24^Ab^	38.53 ± 1.22^Ac^	43.59 ± 0.84^ABa^	42.29 ± 0.80^ABa^	35.48 ± 0.86^ABb^
	T4	38.54 ± 1.48^Ba^	36.25 ± 0.88^BCa^	33.04 ± 1.01^BCb^	41.04 ± 1.20^Ba^	41.25 ± 1.11^ABa^	33.90 ± 1.00^Bb^
2019–2020	T0	36.68 ± 0.89^Ca^	34.04 ± 1.38^Ca^	30.00 ± 1.39^Db^	34.65 ± 1.14^Cb^	38.03 ± 1.07^Ca^	30.53 ± 1.04^Cc^
	T1	39.19 ± 1.05^BCa^	37.21 ± 1.06^BCa^	31.53 ± 1.11^CDb^	42.97 ± 1.28^Ba^	41.82 ± 1.07^Ba^	34.14 ± 0.93^Bb^
	T2	43.02 ± 1.33^Ba^	41.09 ± 1.25^ABa^	36.17 ± 1.06^Bb^	48.44 ± 0.91^Aa^	45.65 ± 0.72^Ab^	38.63 ± 1.00^Ac^
	T3	47.20 ± 1.25^Aa^	45.14 ± 1.36^Aa^	40.81 ± 1.20^Ab^	44.60 ± 0.95^ABa^	43.14 ± 0.99^ABa^	36.78 ± 0.91^ABb^
	T4	40.32 ± 1.08^BCa^	39.22 ± 1.13^Ba^	35.33 ± 0.95^BCb^	42.95 ± 1.13^Ba^	42.35 ± 1.18^ABa^	35.17 ± 1.09^ABb^
		**Mn (mg·kg^−1^)**			**Cu (mg·kg^−1^)**		
2018–2019	T0	29.76 ± 0.77^Ca^	29.17 ± 0.80^Da^	26.36 ± 0.83^Bb^	7.38 ± 0.59^Aa^	7.43 ± 0.62^Aa^	6.39 ± 0.54^Aa^
	T1	32.07 ± 0.71^Ca^	30.72 ± 0.70^CDa^	27.49 ± 0.71^Bb^	6.92 ± 0.52^Aa^	6.94 ± 0.50^Aa^	5.66 ± 0.72^Aa^
	T2	36.44 ± 0.87^Ba^	33.35 ± 0.83^BCb^	28.24 ± 0.75^ABc^	6.93 ± 0.51^Aa^	6.92 ± 0.43^Aa^	5.72 ± 0.73^Aa^
	T3	41.01 ± 1.16^Aa^	36.55 ± 0.87^Ab^	30.43 ± 0.97^Ac^	6.81 ± 0.43^Aa^	6.89 ± 0.50^Aab^	5.59 ± 0.54^Ab^
	T4	35.46 ± 0.71^Ba^	34.33 ± 0.67^ABa^	26.71 ± 0.71^Bb^	5.59 ± 0.53^Ba^	5.52 ± 0.71^Ba^	4.69 ± 0.53^Ba^
							
2019–2020	T0	30.86 ± 1.27^Ca^	30.53 ± 0.88^Ca^	25.88 ± 1.17^Cb^	6.72 ± 0.61^Aa^	6.77 ± 0.62^Aa^	5.77 ± 0.35^Ab^
	T1	34.14 ± 0.81^BCa^	33.33 ± 1.04^BCa^	27.80 ± 0.98^BCb^	6.05 ± 0.56^Aa^	6.10 ± 0.43^Aa^	5.17 ± 0.44^Aa^
	T2	37.62 ± 0.61^Ba^	35.32 ± 1.00^ABb^	30.15 ± 0.95^ABc^	5.91 ± 0.50^Aa^	5.95 ± 0.48^Aa^	5.07 ± 0.61^Aa^
	T3	41.54 ± 1.08^Aa^	38.10 ± 0.96^Ab^	32.15 ± 0.96^Ac^	5.91 ± 0.43^Aa^	5.57 ± 0.61^Aab^	4.84 ± 0.45^Ab^
	T4	36.93 ± 1.09^Ba^	35.41 ± 0.91^ABa^	28.55 ± 1.02^ABCb^	5.07 ± 0.52^Aa^	4.99 ± 0.32^Aa^	4.04 ± 0.61^Aa^

*Different capital letters in the same column indicate a significant difference at α = 0.01. Different lowercase letters in the same row indicate a significant difference at α = 0.05. T0, no Se fertilizer; T1, 1,080 g·ha^−1^ pure Se; T2, 2,160 g·ha^−1^ pure Se; T3, 3,240 g·ha^−1^ pure Se; T4, 4,320 g·ha^−1^ pure Se; BGW-1, black-grained wheat genotype Xihei 88; BGW-2, black-grained wheat genotype Heidali; WGW-2, white-grained wheat cultivar Pubing 151*.

### Effect of Se Application on Pb, As, Hg, and Cr Concentrations in the Grains of Wheat

Pb, As, Hg, and Cr concentrations in the grains of BGW and WGW were in the range of 0.04–0.06, 0.02–0.04, 0.001–0.002, and 0.04–0.06 mg·kg^−1^, respectively, under Se application treatments in the 2019–2020 season (Table 4). There were no significant differences in Pb, As, Hg, or Cr concentrations in grains between T0 and Se application treatments. Regardless of wheat genotypes and the application rates of Se ore powder, Pb, As, Hg, and Cr concentrations in grains were not significantly different.

**Table 4:** Effects of Se fertilization on Pb, As, Hg, and Cr concentrations in grains in the 2019–2020 season.

**Elements**	**Treatments**	**BGW-1**	**BGW-2**	**WGW-2**
Pb (mg·kg^−1^)	T0	0.04 ± 0.01^Aa^	0.03 ± 0.01^Aa^	0.03 ± 0.01^Aa^
	T1	0.05 ± 0.01^Aa^	0.04 ± 0.01^Aa^	0.04 ± 0.01^Aa^
	T2	0.05 ± 0.02^Aa^	0.06 ± 0.02^Aa^	0.05 ± 0.01^Aa^
	T3	0.06 ± 0.02^Aa^	0.05 ± 0.01^Aa^	0.06 ± 0.02^Aa^
	T4	0.06 ± 0.01^Aa^	0.06 ± 0.02^Aa^	0.05 ± 0.01^Aa^
				
As (mg·kg^−1^)	T0	0.02 ± 0.01^Aa^	0.02 ± 0.01^Aa^	0.02 ± 0.01^Aa^
	T1	0.03 ± 0.01^Aa^	0.03 ± 0.01^Aa^	0.03 ± 0.01^Aa^
	T2	0.02 ± 0.01^Aa^	0.03 ± 0.01^Aa^	0.03 ± 0.01^Aa^
	T3	0.03 ± 0.01^Aa^	0.04 ± 0.01^Aa^	0.03 ± 0.01^Aa^
	T4	0.04 ± 0.01^Aa^	0.03 ± 0.01^Aa^	0.04 ± 0.01^Aa^
				
Hg (mg·kg^−1^)	T0	0.001 ± 0.001^Aa^	0.001 ± 0.001^Aa^	0.001 ± 0.001^Aa^
	T1	0.001 ± 0.001^Aa^	0.001 ± 0.001^Aa^	0.001 ± 0.001^Aa^
	T2	0.001 ± 0.001^Aa^	0.001 ± 0.001^Aa^	0.001 ± 0.001^Aa^
	T3	0.002 ± 0.000^Aa^	0.002 ± 0.001^Aa^	0.002 ± 0.000^Aa^
	T4	0.002 ± 0.001^Aa^	0.002 ± 0.000^Aa^	0.002 ± 0.001^Aa^
				
Cr (mg·kg^−1^)	T0	0.04 ± 0.01^Aa^	0.05 ± 0.01^Aa^	0.04 ± 0.01^Aa^
	T1	0.05 ± 0.01^Aa^	0.04 ± 0.01^Aa^	0.06 ± 0.02^Aa^
	T2	0.06 ± 0.01^Aa^	0.05 ± 0.01^Aa^	0.05 ± 0.01^Aa^
	T3	0.05 ± 0.01^Aa^	0.06 ± 0.01^Aa^	0.05 ± 0.01^Aa^
	T4	0.06 ± 0.01^Aa^	0.06 ± 0.01^Aa^	0.06 ± 0.01^Aa^

*Different capital letters in the same column indicate a significant difference at α = 0.01. Different lowercase letters in the same row indicate a significant difference at α = 0.05. T0, no Se fertilizer; T1, 1,080 g·ha^−1^ pure Se; T2, 2,160 g·ha^−1^ pure Se; T3, 3,240 g·ha^−1^ pure Se; T4, 4,320 g·ha^−1^ pure Se; BGW-1, black-grained wheat genotype Xihei 88; BGW-2, black-grained wheat genotype Heidali; WGW-2, white-grained wheat cultivar Pubing 151*.

### Effect of Se Application on Se Uptake in Wheat

Regardless of wheat genotype, the application of Se ore powder significantly increased the uptake of Se in the different parts of the plant (grain, spike-stalk + glum, leaves, stem + sheath, and root) in both seasons (α = 0.01) compared with T0 (Table 5). Se concentration in each part of the wheat plant increased with increasing Se application levels (T1–T4 treatments).

**Table 5:** Effects of Se fertilization on Se concentrations in different plant parts and the translocation factor from the root to the grain of wheat in the two seasons.

**Seasons**	**Treatments**	**Grain (mg·kg^−1^)**			**Spike-stalk + glum (mg·kg^−1^)**			**Leaves (mg·kg^−1^)**		
		**BGW-1**	**BGW-2**	**WGW-2**	**BGW-1**	**BGW-2**	**WGW-2**	**BGW-1**	**BGW-2**	**WGW-2**
2018–2019	T0	0.06 ± 0.01^Ea^	0.06 ± 0.01^Ea^	0.04 ± 0.01^Ea^	0.11 ± 0.01^Ea^	0.10 ± 0.01^Eab^	0.09 ± 0.01^Eb^	0.14 ± 0.01^Ea^	0.13 ± 0.01^Ea^	0.10 ± 0.01^Eb^
	T1	0.23 ± 0.02^Da^	0.21 ± 0.01^Da^	0.15 ± 0.01^Db^	0.29 ± 0.01^Da^	0.27 ±0.01^Dab^	0.25 ± 0.01^Db^	0.41 ± 0.02^Da^	0.38 ± 0.01^Db^	0.34 ± 0.01^Dc^
	T2	0.45 ± 0.03^Ca^	0.42 ± 0.01^Ca^	0.28 ± 0.01^Cb^	0.54 ± 0.04^Ca^	0.51 ± 0.03^Cab^	0.45 ± 0.02^Cb^	0.74 ± 0.02^Ca^	0.69 ± 0.01^Cb^	0.56 ± 0.01^Cc^
	T3	0.68 ± 0.01^Ba^	0.61 ± 0.01^Bb^	0.47 ± 0.01^Bc^	0.78 ± 0.04^Ba^	0.75 ± 0.02^Ba^	0.67 ± 0.01^Bb^	1.29 ± 0.04^Ba^	1.20 ± 0.01^Bb^	1.00 ± 0.01^Bc^
	T4	1.05 ± 0.01^Aa^	0.95 ± 0.01^Ab^	0.73 ± 0.02^Ac^	1.14 ± 0.01^Aa^	1.06 ± 0.01^Ab^	0.93 ± 0.03^Ac^	2.02 ± 0.02^Aa^	1.89 ± 0.01^Ab^	1.70 ± 0.02^Ac^
										
2019–2020	T0	0.07 ± 0.01^Ea^	0.06 ± 0.01^Ea^	0.04 ± 0.01^Ea^	0.10 ± 0.01^Ea^	0.10 ± 0.01^Ea^	0.10 ± 0.01^Ea^	0.15 ± 0.01^Ea^	0.12 ± 0.01^Eb^	0.10 ± 0.01^Ec^
	T1	0.24 ± 0.03^Da^	0.22 ± 0.01^Da^	0.14 ± 0.01^Db^	0.33 ± 0.01^Da^	0.29 ±0.02^Dab^	0.27 ± 0.02^Db^	0.44 ± 0.01^Da^	0.42 ± 0.01^Da^	0.36 ± 0.01^Db^
	T2	0.43 ± 0.01^Ca^	0.40 ± 0.02^Ca^	0.31 ± 0.01^Cb^	0.55 ± 0.01^Ca^	0.52 ± 0.01^Ca^	0.48 ± 0.02^Cb^	0.77 ± 0.01^Ca^	0.72 ± 0.01^Ca^	0.61 ± 0.03^Cb^
	T3	0.71 ± 0.01^Ba^	0.65 ± 0.01^Bb^	0.50 ± 0.02^Bc^	0.82 ± 0.01^Ba^	0.81 ± 0.01^Ba^	0.62 ± 0.01^Bb^	1.25 ± 0.02^Ba^	1.17 ± 0.01^Bb^	0.92 ± 0.02^Bc^
	T4	1.08 ± 0.02^Aa^	1.00 ± 0.02^Ab^	0.78 ± 0.01^Ac^	1.17 ± 0.01^Aa^	1.11 ± 0.01^Ab^	0.96 ± 0.01^Ac^	1.98 ± 0.01^Aa^	1.95 ± 0.01^Aa^	1.65 ± 0.05^Ab^
		**Stem + leaf sheath (mg·kg^−1^)**			**Root (mg·kg^−1^)**			**TF_root−grain_**		
2018–2019	T0	0.12 ± 0.01^Da^	0.11 ± 0.01^Ea^	0.07 ± 0.01^Eb^	0.22 ± 0.02^Ea^	0.23 ± 0.01^Ea^	0.24 ± 0.01^Ea^	0.29 ± 0.01^Ba^	0.26 ± 0.02^Aa^	0.16 ± 0.02^Bb^
	T1	0.28 ± 0.01^Ca^	0.26 ± 0.02^Da^	0.18 ± 0.01^Db^	0.73 ± 0.02^Dc^	0.81 ± 0.01^Db^	0.88 ± 0.01^Da^	0.31 ± 0.03^Ba^	0.26 ± 0.01^Ab^	0.17 ± 0.01^Bc^
	T2	0.55 ± 0.02^Ba^	0.43 ± 0.01^Cb^	0.35 ± 0.01^Cc^	1.45 ± 0.03^Cb^	1.56 ± 0.01^Ca^	1.61 ± 0.02^Ca^	0.31 ± 0.01^Ba^	0.27 ± 0.01^Ab^	0.17 ± 0.01^Bc^
	T3	0.66 ± 0.02^Ba^	0.58 ± 0.02^Bb^	0.51 ± 0.02^Bc^	1.87 ± 0.03^Bc^	2.02 ± 0.01^Bb^	2.12 ± 0.01^Ba^	0.36 ± 0.01^Aa^	0.30 ± 0.01^Ab^	0.22 ± 0.01^Ac^
	T4	1.11 ± 0.01^Aa^	1.05 ± 0.05^Aa^	0.85 ± 0.01^Ab^	2.72 ± 0.03^Ac^	2.83 ± 0.05^Ab^	2.94 ± 0.02^Aa^	0.39 ± 0.01^Aa^	0.34 ± 0.02^Ab^	0.25 ± 0.01^Ac^
										
2019–2020	T0	0.11 ± 0.01^Ea^	0.10 ± 0.01^Ea^	0.08 ± 0.01^Eb^	0.23 ± 0.02^Eb^	0.25 ± 0.01^Dab^	0.28 ± 0.01^Ea^	0.31 ± 0.01^Ba^	0.24 ± 0.02^Ca^	0.14 ± 0.01^Cb^
	T1	0.32 ± 0.02^Da^	0.25 ± 0.03^Db^	0.21 ± 0.01^Db^	0.80 ± 0.01^Db^	0.84 ± 0.07^Cb^	0.97 ± 0.01^Da^	0.30 ± 0.02^Ba^	0.26 ±0.03^BCa^	0.14 ± 0.01^Cb^
	T2	0.52 ± 0.01^Ca^	0.42 ± 0.01^Cb^	0.38 ± 0.01^Cc^	1.40 ± 0.04^Cb^	1.61 ± 0.05^Ba^	1.71 ± 0.01^Ca^	0.31 ± 0.02^Ba^	0.25 ± 0.01^Cb^	0.18 ± 0.01^BCc^
	T3	0.70 ± 0.01^Ba^	0.61 ± 0.01^Bb^	0.57 ± 0.02^Bc^	1.92 ± 0.03^Bc^	2.09 ± 0.01^Bb^	2.19 ± 0.01^Ba^	0.37 ± 0.01^Aa^	0.31 ±0.01^ABb^	0.23 ± 0.01^ABc^
	T4	1.13 ± 0.05^Aa^	1.04 ± 0.03^Aa^	0.90 ± 0.02^Ab^	2.68 ± 0.16^Ab^	2.81 ± 0.03^Aab^	2.97 ± 0.03^Aa^	0.40 ± 0.03^Aa^	0.35 ± 0.01^Ab^	0.26 ± 0.01^Ac^

*Different capital letters in the same column indicate a significant difference at α = 0.01. Different lowercase letters in the same row indicate a significant difference at α = 0.05. T0, no Se fertilizer; T1, 1,080 g·ha^−1^ pure Se; T2, 2,160 g·ha^−1^ pure Se; T3, 3,240 g·ha^−1^ pure Se; T4, 4,320 g·ha^−1^ pure Se; BGW-1, black-grained wheat genotype Xihei 88; BGW-2, black-grained wheat genotype Heidali; WGW-2, white-grained wheat cultivar Pubing 151*.

The effects of Se application on Se concentration in the different parts of the plant were compared between BGW (BGW-1 and BGW-2) and WGW (WGW-2) under the treatments (Table 5). Se concentrations in grains, spike-stalk + glum, leaves, and stem + sheath of BGW-1 were significantly higher than those of WGW-2 under T1, T2, T3, and T4 treatments, but Se concentration in roots was significantly lower than that in WGW-2 (α = 0.05). Compared with WGW-2, BGW-2 had significantly higher Se concentration in grains and leaves under T1, T2, T3, and T4 treatments and also had the same Se concentration in stem + leaf sheath under CK, T2, T3, and T4 treatments, but Se concentration in roots was significantly lower than that in WGW-2 under T1 and T3 treatments (α = 0.05).

Table 5 also shows the translocation factor of Se from the root to grain (TF_root−grain_) of wheat genotypes. The application of Se ore powder affected TF_root−grain_. BGW-1 and WGW-2 had a significantly higher TF_root−grain_ under T3 and T4 treatments in comparison to CK (α = 0.01). A significantly higher TF_root−grain_ was found for BGW (α = 0.05) under the treatments compared with WGW-2.

### Effect of Se Application on Se Distribution in Wheat

**Figure 2:**
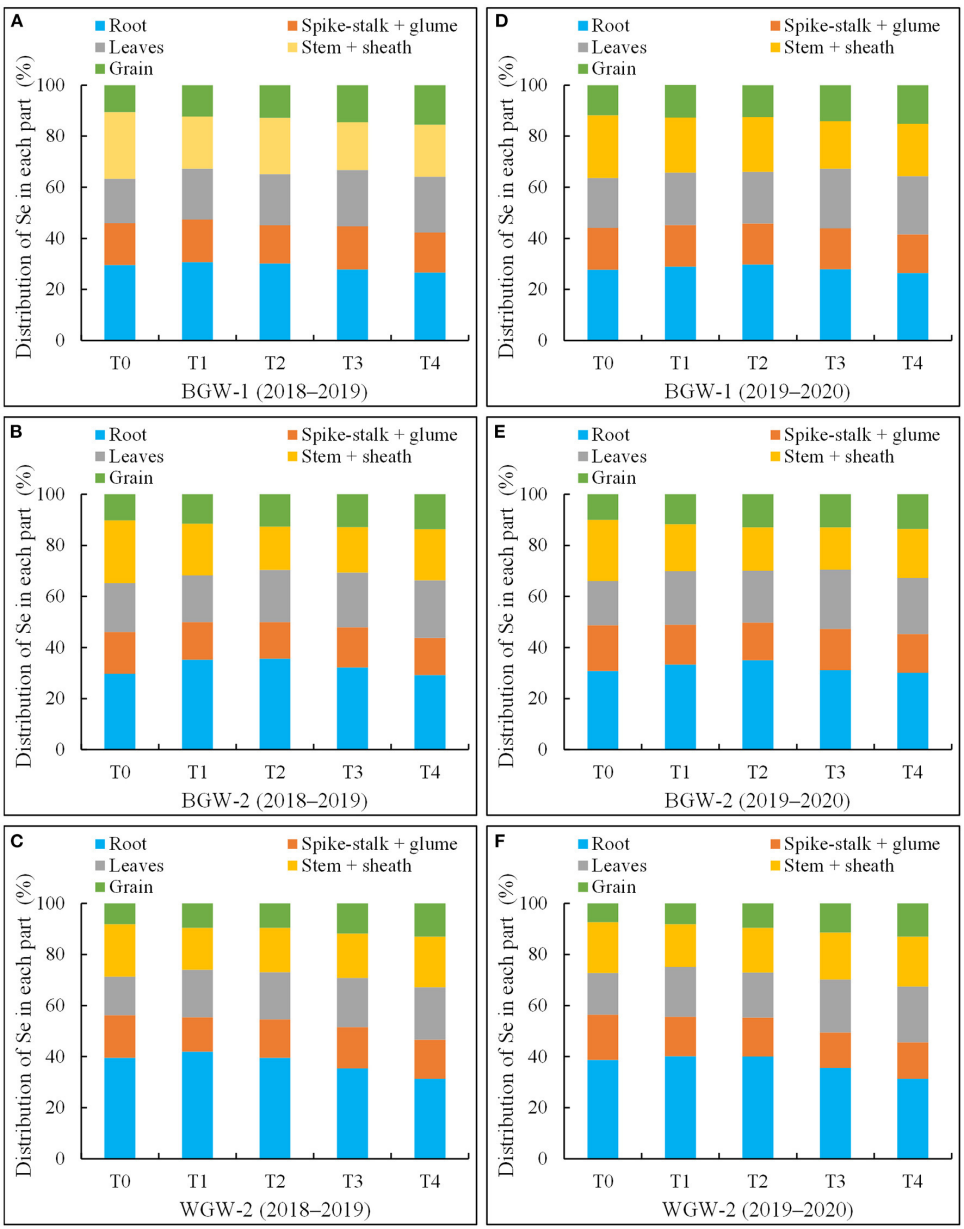
The percentages of selenium (Se) in roots, spike-stalk + glum, leaves, stem + sheath, and grains compared to that of total Se in black-grained wheat (BGW) (BGW-1 and BGW-2) and white-grained wheat (WGW) (WGW-2) genotypes in the two growing seasons. Se distribution in each part represents the proportion in the total Se taken up by the whole plant. T0, no Se fertilizer; T1, 1,080 g·ha^−1^ pure Se; T2, 2,160 g·ha^−1^ pure Se; T3, 3,240 g·ha^−1^ pure Se; T4, 4,320 g·ha^−1^ pure Se; BGW-1, BGW genotype Xihei 88; BGW-2, BGW genotype Heidali; WGW-2, WGW cultivar Pubing 151. **(A–F)**, the distribution of Se in each plant of BGW-1 (2018–2019), BGW-2 (2018–2019), WGW-2 (2018–2019), BGW-1 (2019–2020), BGW-2 (2019–2020), and WGW-2 (2019–2020), respectively.

Selenium distribution and accumulation in the different parts of wheat were affected by the application of Se ore powder (Figure 2). A large amount of Se was distributed in roots under all treatments in both growing seasons, whereas a small proportion of the total Se was found in grains. Se distribution in roots was first increased and then reduced with increasing Se application levels, whereas Se distribution in grains was increased.

The roots of BGW-1 (26.6–30.7% in the 2018–2019 season and 26.4–29.8% in the 2019–2020 season) had a significantly lower percentage of Se than that of WGW-2 (31.7–41.9% in the 2018–2019 season, and 32.2–40.1% in the 2019–2020 season) under all treatments, but the grains of BGW-2 (10.6–15.5% in the 2018–2019 season and 11.8–15.1% in the 2019–2020 season) had a significantly higher percentage of Se than that of WGW-2 (8.1–12.7% in the 2018–2019 season and 7.4–12.5% in the 2019–2020 season) (α = 0.05), due to the possession of higher uptake ability by BGW-1 for the soil application of Se ore powder (Table 5).

### Effect of Se Application on Organic Se in the Grains of Wheat

The application of Se ore powder significantly increased the organic Se concentration in wheat grains, compared with CK (α = 0.01) (Table 6). The organic Se concentration was significantly increased with increasing Se application levels (T1–T4 treatments) (α = 0.01).

**Table 6:** Effects of Se fertilization on organic Se concentration in the grain of wheat in the two growing seasons.

**Seasons**	**Treatments**	**Organic Se (mg·kg^−1^)**		
		**BGW-1**	**BGW-2**	**WGW-2**
2018–2019	T0	0.05 ± 0.00 (76.3%)^Ea^	0.04 ± 0.00 (74.8%)^Ea^	0.03 ± 0.00 (70.8%)^Eb^
	T1	0.18 ± 0.01 (77.1%)^Da^	0.16 ± 0.01 (76.4%)^Da^	0.11 ± 0.01 (72.5%)^Db^
	T2	0.35 ± 0.02 (78.6%)^Ca^	0.33 ± 0.02 (78.5%)^Ca^	0.21 ± 0.01 (76.4%)^Cb^
	T3	0.55 ± 0.03 (80.5%)^Ba^	0.48 ± 0.02 (79.2%)^Bb^	0.37 ± 0.03 (79.2%)^Bc^
	T4	0.85 ± 0.03 (81.4%)^Aa^	0.78 ± 0.03 (81.7%)^Ab^	0.60 ± 0.03 (79.4%)^Ac^
				
2019–2020	T0	0.05 ± 0.01 (76.0%)^Ea^	0.04 ± 0.01 (74.5%)^Eab^	0.03 ± 0.00 (70.0%)^Eb^
	T1	0.19 ± 0.01 (78.4%)^Da^	0.17 ± 0.01 (76.7%)^Db^	0.13 ± 0.01 (74.4%)^Dc^
	T2	0.34 ± 0.02 (79.7%)^Ca^	0.31 ± 0.02 (77.8%)^Ca^	0.24 ± 0.01 (78.1%)^Cb^
	T3	0.58 ± 0.03 (81.2%)^Ba^	0.53 ± 0.03 (80.8%)^Bb^	0.39 ± 0.02 (77.8%)^Bc^
	T4	0.89 ± 0.03 (82.2%)^Aa^	0.81 ± 0.03 (81.0%)^Ab^	0.63 ± 0.03 (78.8%)^Ac^

*Different capital letters in the same column indicate a significant difference at α = 0.01. Different lowercase letters in the same row indicate a significant difference at α = 0.05. T0, no Se fertilizer; T1, 1,080 g·ha^−1^ pure Se; T2, 2,160 g·ha^−1^ pure Se; T3, 3,240 g·ha^−1^ pure Se; T4, 4,320 g·ha^−1^ pure Se; BGW-1, black-grained wheat genotype Xihei 88; BGW-2, black-grained wheat genotype Heidali; WGW-2, white-grained wheat cultivar Pubing 151. The percent was the ratio of organic Se to total Se in the grain*.

The grains of BGW (BGW-1 and BGW-2) had significantly higher organic Se concentrations than those in WGW (WGW-2) under Se application treatments (α = 0.05). The grains of BGW-1 (76.3–81.4% in the 2018–2019 season and 76.0–82.2% in the 2019–2020 season) had a higher percentage of organic Se in total Se than those of WGW-2 (70.8–79.4% in the 2018–2019 season and 70.0–78.8% in the 2019–2020 season) under all treatments in both seasons.

## Discussion

### Wheat Grown in Se-Rich Areas Increases Its Grain Yield and Crude Protein Content

In addition to being essential for humans, Se is beneficial for plants in antioxidant activity/response and as a growth promoter (Galic et al., 2021). Plants play an important role in overcoming Se deficiency and Se toxicity in the different regions of the world (Gupta and Gupta, 2007). The uptake of Se by plants is governed by multiple factors. Among the determining factors, the most important factor is the concentration and form of Se in soils. The average concentration of Se was 5.70 mg·kg^−1^ in Daba mountain soil, and Se concentration was positively corrected with Ca, Mg, P, and Zn concentration (Li et al., 2008). Se fractions in soils include sulfide/selenide and base-soluble Se around Se-rich cores in Ziyang Country (Zhang et al., 2018). Species of soluble Se in upland soil from Naore Village, Ziyang, China included selenide (Se^−2^), Se^4+^, and Se^6+^, and soluble Se^6+^ directly affected Se accumulation in corn tissues (Wang et al., 2012). Se^4+^ is the main Se valence, and almost no Se^6+^ was measured in soils in which paddy rice was planted, whereas a nearly 1:1 ratio of Se^4+^ and Se^6+^ existed for soils in which maize was planted (Zhang et al., 2018). Se^4+^ can be easily taken up and transported by the xylem (Zhou et al., 2020). The concentration of Se in plant tissues grown in a Shuang‘an site was increased by artificial disposal of ore dumps, waste rocks, and weathering of *in situ* seleniferous rock (Fang and Wu, 2004). The distribution trend of Se concentration in the grains of wheat was consistent with the distribution of the available Se concentration in soils (Fang and Wu, 2004).

In this study, the results showed that Se concentration in wheat (black- and white-grained) grains were significantly decreased from high Se (Shuang ‘an) to low Se areas (Yangling) (α = 0.01) (Table 1), which was consistent with the study by Fang and Wu (2004). A higher grain yield of wheat was found in the Se-rich areas (Se concentration in the soil with 2.02–2.21 mg·kg^−1^) (Table 1). This result may be caused due to a role played by Se in plant growth (Galic et al., 2021) and the protection of photosynthetic capacity in plants, and Se is considered to be an advantageous element for plant resistance to abiotic stresses, resulting in high grain yield (Gupta and Gupta, 2007). Interestingly, higher grain Se concentration and crude protein content were also significantly observed for BGW-1 in the Se-rich areas (α = 0.05) compared with WGW (Table 1). This was due to the pigmented wheat cultivars having higher Se efficiency absorption and Se flow direction, mainly increasing the concentration of Se-rich protein of low molecular weight glutenin subunits (LMW-GS) (Pu et al., 2021). In addition, crude fat and ash contents were unaffected. Based on these results, wheat planting directly into soils with naturally abundant Se could be a more efficient and effective option for producing high-yield, Se-enriched, and nutritious wheat foods.

### Soil Application of Se Ore Powder Improves the Plant Growth and Grain Yield of Wheat

Generally, Se in rocks represents the major source for Se in soils, and soils with Se have a much greater effect on Se reinforcement in crops. Most regions have low Se concentration, but Enshi City and Ziyang County are the two notable seleniferous regions in China. The application of Se-rich rocks has been reported in farming practices. In Enshi, Se-rich products such as stone coal were mined as a fuel or fertilizer and applied to croplands to improve the soil (Zhu et al., 2008). Se-enriched slate as the Se fertilizer collected from Ziyang was used to plant potatoes (Tian et al., 2016a). Se ore powder from Enshi was applied to maize, rice, and soybean fields before planting (Liu et al., 2016a; Deng et al., 2018). Liu et al. (2016a) reported that the application of Se mineral powder (180 mg·kg^−1^ pure Se) with different levels (750, 1,500, 2,250, and 3,000 g·ha^−1^) had no significant effects on the biomass or grain yield of maize, but Se concentrations in maize grains and maize organs were improved significantly. Deng et al. (2018) showed that the application of Se ore powder at a rate of 0.3 t·ha^−1^ (747.5 g·ha^−1^ pure Se) had a slight but nonsignificant stimulatory effect on the grain yield and total biomass of both rice and soybeans. However, soil application with Se-rich solid fertilizer (37.5 g·ha^−1^ as the form of Se^4+^) increased the growth of wheat, resulting in higher shoot dry weight and grain yield (Xia et al., 2020). (Wang et al., 2021) showed that regardless of the Se rate, the soil application of both Se^4+^ and Se^6+^ decreased the biomass and grain yield in almost all wheat cultivars, whereas Boldrin et al. (2013) reported that the soil application of Se^4+^ or Se^6+^ had no effect on the growth of rice. De Vita et al. (2017) showed that foliar application of Se^6+^ had no effect on the growth of wheat, whereas Boldrin et al. (2013) concluded that the soil application of Se^4+^ or Se^6+^ promotes the growth of rice. The available data suggest that the beneficial effects of the application of Se on plant growth and yield depend on several factors, such as application methods, Se sources, Se application dose, crop types, and plant growth conditions.

In this study, the results showed that the soil application of Se mineral powder (1,080, 2,160, 3,240, and 4,320 g·ha^−1^) increased the grain yield, biomass, harvest index, grain number, and 1,000 kernels weight of wheat, and the greatest effect was observed when Se was applied at 2,160 g·ha^−1^ (Table 2). However, such a beneficial effect was not found for spike number. The differences may have been caused by application methods. Soil application in this study was done before tilling, where the effect of Se supplementation could be made through Se uptake by roots. The observed increases in grain yield and its components of wheat might be due to certain minerals in Se ore powder promoting the growth of wheat.

### Is It Safe and Feasible to Apply Se Ore Powder From Ziyang to Produce Se-Rich Wheat Products?

The application of Se ore powder increased the absorption of Zn, Mn, and Cu in the grains of maize at certain application doses (Liu et al., 2016a). Foliar application Se (Na_2_SeO_3_) increased the concentrations of Fe and Zn in the grains of colored-grain wheat but decreased the concentrations of Cu and Mn (Xia et al., 2019). In this study, Zn, Fe, and Mn concentrations in the grains of wheat were first increased and then decreased with an increase of Se application, whereas Cu concentration was decreased (Table 3), indicating that the soil application of Se ore powder has a positive effect on Zn, Fe, and Mn concentrations in the grains of wheat. Interestingly, the highest Zn and Mn value was observed under T3 treatment (3,240 g·ha^−1^ pure Se), whereas the value of Fe was the highest under T2 treatment (2,160 g·ha^−1^ pure Se), suggesting that the absorption capacity of wheat for Zn, Mn, and Fe varies with the applied dose of Se ore powder.

When Se-rich rocks are used to fertilize the soils, serious consideration should be given to heavy metal contamination related to Se enrichment. Deng et al. (2018) reported that As, Hg, Cr, and Pb concentrations in Se ore powder from Enshi were lower than the soil background values in most cases. Tian et al. (2016b) found that Cd, V, As, and Pb were highly enriched in Se-rich stone coal and rocks in Ziyang. Food safety assessment is essential to determine whether Se ore powder can be directly used to produce Se-rich agricultural products. In this study, we found that Pb, As, Hg, and Cr concentrations in BGW and WGW grains were lower than the Limits of Contaminants in Food, National Food Safety Standard in China (GB 2762-2017) (Pb ≤ 0.2 mg·kg^−1^, As ≤ 0.5 mg·kg^−1^, Hg ≤ 0.02 mg·kg^−1^, and Cr ≤ 0.1 mg·kg^−1^ in cereal grains), the following application of Se ore powder, which was consistent with the findings of Deng et al. (2018). Previous studies suggest that the applied Se ore powder from Ziyang is soft and feasible.

The distribution of Se in the different parts of plants varies according to crop species, the stages of plant development, and plant physiological condition (Terry et al., 2000). Deng et al. (2018) reported that the application of Se ore powder significantly increased Se concentration in various parts of rice and soybeans, and Se concentration in the different parts of their plants was in the order of root > straw > brown rice > husk and seed > root > straw > pod. Liu et al. (2016a) reported the Se accumulation ability in different maize organs descended in the order of root > leaf > grain > stem. Wang et al. (2021) showed that the soil application of both Se^4+^ and Se^6+^ treatments increased the uptake of Se in each part of the wheat plant. Wheat is considered a non-accumulating cereal crop as it often shows approximately the same Se concentration in grains and roots and smaller amounts in the stem and leaves at the mature stage (Terry et al., 2000). However, more Se accumulation was observed in the grains and roots of wheat with Se^4+^ application while Se was mostly accumulated in the leaves and straw with Se^6+^ treatment (Wang et al., 2021). In this study, Se concentration in the different parts of wheat was also increased by the soil application of Se ore powder. More Se accumulation was observed in roots with Se ore powder, whereas a smaller amount of total Se was found in grains.

More bioavailability has been recognized in organic Se species than in inorganic Se species. Liu et al. (2016a) showed that the total Se and organic Se concentration in grains were significantly increased with the increasing application rates of Se ore powder, but no significant differences were found for the ratio of organic Se to total Se in grains. In this study, similar results for the total Se and organic Se concentration in grains were also found when applying Se ore powder from Ziyang to the soil, but one strain of BGW (BGW-1) had a higher ratio of organic Se to total Se in grains than the WGW under different Se treatments, indicating that the conversion capacity of total Se to organic Se differs according to the colored-grain wheat genotype.

Based on these results, Se ore powder from Ziyang as Se fertilization could be a promising strategy for Se biofortification in wheat.

### Higher Sensitivity to Se Accumulation in BGW Genotypes Than in the WGW Genotype

Pu et al. (2021) showed that the quality of the pigmented wheat under Se treatment was more improved than others. Xia et al. (2020) reported that the purple-grained wheat 202w17 accumulated more Fe, Zn, Ca, and Mg in grains than the common wheat Shannong 129 when Se was applied as a foliar spray. The accumulation of total Se in shoots and grains and organic Se in grains was also observed in the purple-grained wheat 202w17 following the soil application and foliar spray of Se (Xia et al., 2020). In this study, Zn, Fe, and Mn concentrations in the grains of BGW genotypes (BGW-1 and BGW-2) were significantly higher than those of the WGW BGW-1 under different treatments (Table 3), which were in contrast to the study of Xia et al. (2020). High Se occurs in association with high Zn and Fe in some genotypes. Therefore, BGW genotypes may be the most desirable genetic resources for biofortification.

This study showed that when Se ore powder was applied to the soil, BGW genotypes (BGW-1 and BGW-2) had a higher accumulation of Se in grains and shoots and more organic Se in grains than the WGW (WGW-2), but a lower accumulation of Se was found in roots (Table 5; Figure 2), suggesting that BGW can translocate the absorbed Se to the grain more easily than the WGW. The results were in agreement with the findings by Xia et al. (2020) and Pu et al. (2021). This may be one of the reasons for the higher crude protein content in BGW genotypes (Table 1). Another reason may be that the pigmented varieties have higher Se^6+^ absorption capacity in the soil because BGW genotypes (BGW-1 and BGW-2) have a higher translocation factor of Se from the root to grain than the WGW (WGW-2) under controlled conditions (Table 5). Based on these results, BGW is potentially the most Se-rich genotype.

## Data Availability Statement

The datasets presented in this study can be found in online repositories. The names of the repository/repositories and accession number(s) can be found in the article/supplementary material.

## Author Contributions

YL and ZZ conceived and designed the study. YL, SH, ZJ, YW, and ZZ performed the experiments. YL, SH, ZJ, and ZZ analyzed the data, prepared figures and/or tables, and wrote this article. All authors contributed to this article and approved the submitted version.

## Funding

This research was funded by the National Key Research and Development Program of China (2016YFD0102004), the Key Research and Development Project of Shaanxi Province (No. 2019ZDLNY04-05), Science and Technology Plan Project of Xi'an City (20193043YF031NS031), and start-up funds from Northwest A&F University.

## Conflict of Interest

The authors declare that the research was conducted in the absence of any commercial or financial relationships that could be construed as a potential conflict of interest.

## Publisher's Note

All claims expressed in this article are solely those of the authors and do not necessarily represent those of their affiliated organizations, or those of the publisher, the editors and the reviewers. Any product that may be evaluated in this article, or claim that may be made by its manufacturer, is not guaranteed or endorsed by the publisher.
